# Regulation and clinical potential of telomerase reverse transcriptase (TERT/hTERT) in breast cancer

**DOI:** 10.1186/s12964-023-01244-8

**Published:** 2023-08-23

**Authors:** Ruozhu Yang, Yi Han, Xinyu Guan, Yue Hong, Jiahao Meng, Shirong Ding, Qian Long, Wenjun Yi

**Affiliations:** 1 Department of General Surgery, the Second Xiangya Hospital of Central South University, 139 Middle Renmin Road, Changsha, 410011 China; 2 Department of Oncology, the Second Xiangya Hospital of Central South University, 139 Middle Renmin Road, Changsha, 410011 China

**Keywords:** Telomerase reverse transcriptase, Breast cancer, Promoter mutations, Epigenetic modifications, Transcriptional regulation, Biomarker

## Abstract

Telomerase reverse transcriptase (TERT/hTERT) serves as the pivotal catalytic subunit of telomerase, a crucial enzyme responsible for telomere maintenance and human genome stability. The high activation of hTERT, observed in over 90% of tumors, plays a significant role in tumor initiation and progression. An in-depth exploration of hTERT activation mechanisms in cancer holds promise for advancing our understanding of the disease and developing more effective treatment strategies. In breast cancer, the expression of hTERT is regulated by epigenetic, transcriptional, post-translational modification mechanisms and DNA variation. Besides its canonical function in telomere maintenance, hTERT exerts non-canonical roles that contribute to disease progression through telomerase-independent mechanisms. This comprehensive review summarizes the regulatory mechanisms governing hTERT in breast cancer and elucidates the functional implications of its activation. Given the overexpression of hTERT in most breast cancer cells, the detection of hTERT and its associated molecules are potential for enhancing early screening and prognostic evaluation of breast cancer. Although still in its early stages, therapeutic approaches targeting hTERT and its regulatory molecules show promise as viable strategies for breast cancer treatment. These methods are also discussed in this paper.

Video Abstract

**Supplementary Information:**

The online version contains supplementary material available at 10.1186/s12964-023-01244-8.

## Background

Normal human somatic cells have a limited capacity for division in vitro due to cellular senescence, a phenomenon known as the Hayflick limit [1]. The replication of DNA is semi-conservative, resulting in the progressive shortening of chromosome ends with each replication. These shortened segments, called telomeres, play a crucial role in protecting chromosome integrity and can be elongated by binding to telomerase [2]. Activation of telomerase enables the repair of telomeres, ensuring the protection of chromosome ends and extending the lifespan of cells [3]. This is how most cancer cells maintain telomere length. Additionally, in a small proportion of cases, telomere elongation occurs through an alternative mechanism known as alternative lengthening of telomeres (ALT) [4]. This mechanism does not involve the action of telomerase.

Telomerase is a special reverse transcriptase. It consists of telomerase reverse transcriptase (TERT/hTERT), telomerase RNA (TERC), and some accessory proteins [5, 6]. Among them, hTERT serves the crucial role of reverse-transcribing TERC to synthesize telomeric tandem repeats. This is how telomerase functions [7]. In normal adult somatic cells, hTERT is generally not actively expressed, which corresponds to lower telomerase activity. Most cancer cells have shorter telomeres than adjacent normal tissue cells, but these cancer cells are still able to maintain these short telomeres due to higher telomerase activity [2, 8]. High expression of hTERT can be found in most cancer cells, which is an important reason for higher telomerase activity. In a study involving 31 cancer types, hTERT expression was present in 73% of tumors [9]. The mechanisms of hTERT expression in cancer cells include promoter mutations, epigenetic modifications, transcription factors, single nucleotide polymorphisms, alternative splicing, and copy number amplification [10–13]. Highly expressed hTERT mainly plays a role in telomere maintenance. In addition, hTERT can also regulate cell cycle progression, apoptosis, cell adhesion, and migration through a variety of other pathways [14]. At present, there have been many related studies on breast cancer. In addition to the similarities with other cancers, the regulation of hTERT expression in breast cancer also has its characteristics [15]. Furthermore, less-explored regulatory mechanisms, such as long non-coding RNA (lncRNA) and competitive endogenous RNA (ceRNA) interactions, along with post-translational modifications, also contribute to the expression of hTERT in breast cancer.

This review summarizes the various mechanisms of hTERT expression regulation in breast cancer. Furthermore, we explore the potential utility of hTERT as a diagnostic and prognostic biomarker in breast cancer, with a particular focus on the cellular localization of hTERT and the detection of circulating tumor cells expressing hTERT. Insight into the regulatory mechanisms of hTERT expression may inform treatment adjustments and inspire the development of novel therapeutic approaches for breast cancer. Promising therapeutic strategies, including G-quadruplex stabilizers, epigenetic regulatory drugs, agents targeting related signaling pathways, and therapeutic hTERT vaccines, are discussed in brief, providing valuable insights into the clinical significance of TERT activation.

## hTERT regulation mechanism in breast cancer

### hTERT promoter mutations

**Fig. 1:**
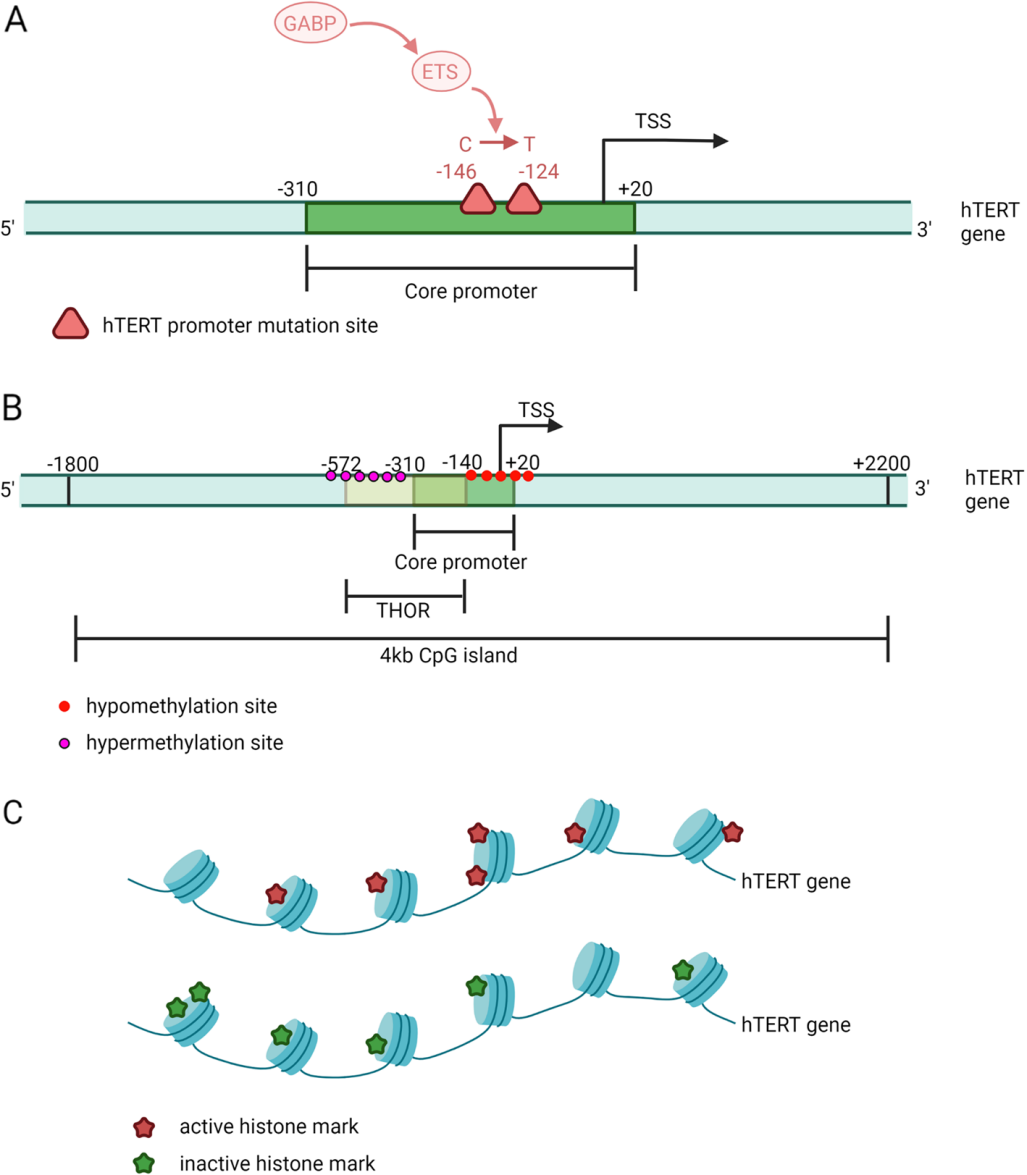
**A** hTERT promoter mutations. The core promoter of hTERT gene is located from -310 to -20 bp of the TSS. Promoter mutations mainly occur at -124 and -146 bp of TSS and result in C to T changes. These mutations generate an EST transcription factor binding motif that recruits GABP to bind to it and subsequently activates hTERT transcription. **B** hTERT DNA methylation. The core promoter of hTERT is located in a 4 kb CpG island from -1800 to + 2200 bp of TSS. Hypermethylation around -600 bp (mainly in the THOR region) of the TSS and hypomethylation around the TSS have been found in various cancer types including breast cancer. **C** hTERT histone mark modifications. DNA is wound around the histone octamer. They form nucleosomes with histone H1 and repeat continuously. A large number of epigenetic marks can be found in the tails of histone proteins. In breast cancer, multiple active histone marks and inactive histone marks have been found. Created with BioRender.com

Mutations in the hTERT promoter (TERTp) are primarily observed at base pairs -124 and -146 relative to its transcription start site, resulting in C to T changes known as C228T and C250T mutations [9, 16, 17] (Fig. 1A). Tumors originating from tissues characterized by high self-renewal rates, such as breast cancer [18] and gastrointestinal malignancies, exhibit a low frequency of TERTp mutations. Conversely, tumors derived from tissues with limited growth potential, such as glioma and thyroid tumors, tend to have a higher occurrence of TERTp mutations [19]. Monoallelic mutations are the predominant form of TERTp mutations [17, 20–22]. Mutations in TERTp generate an EST transcription factor binding motif [21], which then binds to GABP, thereby activating hTERT transcription and telomerase activation, which is linked to disease progression and relapse [23–26] (Fig. 1A).

TERT C228T promoter mutation and 16p13.3 amplification were discovered in a distant metastatic secretory carcinoma of the breast [27]. In invasive ductal carcinoma, -124C > T mutations and -146C > T mutations were found together [28].

hTERT promoter mutation and gene amplification are rare in common breast cancers [29, 30], and are more common in rare phyllodes neoplasms [24, 31]. In a study of 60 metaplastic breast cancers (MBCs) of varying chemical composition, hTERT promoter mutations and gene amplification were less common in MBCs with predominant chondroid differentiation and were mutually exclusive with TP53 mutations [18]. TERT promoter hotspot mutations were found in a substantial subset of non-chondroid MBCs (17%) [18]. Furthermore, in a study involving 28 metaplastic breast cancers, nearly half of the tumors with spindle/squamous differentiation were enriched for TERT promoter mutations [32].

hTERT promoter mutations have the potential to interact with mutations occurring in other genes. In a study, activating mutations in Ras pathway genes, such as KRAS, NRAS, and HRAS Q61R, were identified in three cases of MBCs exhibiting TERT promoter hotspot mutations. Notably, these mutations coexisted with TP53 mutations (subclone V173L) [18]. Another study by Edaise M da Silva et al. demonstrated a negative correlation between TERT promoter hotspot mutations or TERT gene amplification and TP53 mutations, while significant correlations were observed with PIK3CA hotspot mutations in MBCs [18].

### Epigenetic modifications

Epigenetic modifications refer to alterations in DNA activity or function that can be stably inherited without changes to the DNA sequence [33]. These modifications can vary across different tissues and cells, contributing to the development of various diseases, including breast cancer. Recent studies focusing on hTERT epigenetic modifications in breast cancer have explored DNA methylation, histone mark modifications, micro-RNAs, and other mechanisms. DNA methylation and histone mark modification regulate hTERT gene expression at the transcriptional level [33, 34]. Since non-coding RNAs such as micro-RNA and lncRNA regulate hTERT gene expression at the post-transcriptional level, they are also included in epigenetic regulation.

#### hTERT DNA methylation

DNA Methylation is catalyzed by DNA methyltransferase (DNMT). In the process of methylation, the covalent addition of a methyl group to the fifth position of the cytosine residue occurs, which forms 5-methyl cytosine (5mC). These cytosines are located in CpG dinucleotides of CpG islands [35, 36]. CpG islands are mainly located in non-coding regions such as promoters, and about 70% of human genes have promoters with high CpG content [37]. In most cases, the promoter of genes that can be transcribed is unmethylated, and promoter methylation leads to gene silencing. Thus, promoter methylation plays a vital role in establishing tissue-specific gene expression patterns.

Promoter methylation is important in the expression of hTERT in cancer cells. The majority of cancer cells exhibit hTERT expression, suggesting the absence of promoter methylation. The core promoter of hTERT resides within a CpG island spanning approximately 4 kb and is characterized by a high GC content of up to 70%. This CpG island extends from -1800 to + 2200 bp relative to the transcription start site (TSS), with the core promoter specifically located from -310 to -20 bp upstream of the TSS [38]. In the study conducted by Rebekah L. Zinn et al., it was observed that various cancer cell lines, including breast cancer, exhibited hypermethylation approximately -600 bp upstream of the hTERT promoter's transcription start site (TSS), while the regions proximal to the TSS remained unmethylated (Fig. 1B). Notably, unmethylated DNA was associated with active chromatin marks, such as acetyl-H3K9 and dimethyl-H3K4, whereas methylated DNA around the TSS correlated with inactive chromatin marks like trimethyl-H3K9 and trimethyl-H3K27. This finding suggests that despite the hypermethylation around -600 bp, the unmethylated state within the TSS region (-150 to + 150 bp) is critical for facilitating hTERT expression in cancer cells [39].

The question arises: Why does hypermethylation around -600 bp upstream of the transcription start site (TSS) fail to result in the transcriptional repression of hTERT? In 2019, Donghyun D. Lee et al. identified a genomic region spanning 52 CpG sites upstream of the hTERT TSS (-140 to -572 bp), referred to as the TERT hypermethylated oncological region (THOR) (Fig. 1B). Interestingly, the unmethylated state of THOR serves to suppress hTERT promoter activity, while hypermethylation of THOR implies the transcriptional activation of hTERT. THOR is found to be hypermethylated in a majority of human cancer types compared to normal tissues, with a prevalence of up to 75% in breast cancer [40]. Consistent with these findings, Teisha J. Rowland et al. observed hypermethylation in the promoter regions upstream of the TSS and hypomethylation in the proximal promoter regions near the TSS across 23 different types of cancer tissues [41].

Methylation occurring at various sites within the CpG island within the hTERT promoter regulates hTERT expression primarily by influencing the binding of transcription factors to the regulatory sequences of the hTERT gene. Yuanyuan Li et al. demonstrated that genistein downregulates the expression of DNMT, which results in hypomethylation of the binding site for the transcription repressor E2F1 near the transcription start site (TSS). Consequently, enhanced binding of E2F1 to the hTERT promoter leads to the inhibition of hTERT transcription in breast cancer cells [42, 43]. According to Syed M Meeran et al., sulforaphane induces demethylation of the binding site for the transcription factor CTCF. This demethylation promotes CTCF binding to exon 1 of the unmethylated hTERT gene, thereby downregulating hTERT expression in breast cancer cells [44, 45]. Another study by Meeran SM et al. revealed that EGCG and its prodrug inhibit DNMT, reduce promoter methylation (-288 to -31 bp), and enhance the binding of the transcription repressor E2F1 to the hTERT regulatory region. Consequently, hTERT transcription is inhibited in both ER-positive and ER-negative human breast cancer cells [43, 46]. Aloe-emodin induces demethylation of multiple CpG sites ranging from -238 to 65 bp in the hTERT gene promoter, including the binding sites for c-MYC and E2F1. This demethylation leads to decreased hTERT mRNA and protein levels in MDA-MB-453 and MCF-7 breast cancer cells. Additionally, it is accompanied by the activation of the transcription factor E2F1 and inactivation of c-MYC [47]. Thus, it is likely that the impact of hTERT promoter methylation on transcription factor binding is also implicated in this study. Recent investigations on THOR have revealed significantly higher levels of hypermethylation in breast cancer tissues compared to benign tissues. THOR serves as an important epigenetic marker in breast carcinogenesis and holds promise as a biomarker and therapeutic target for early diagnosis of breast cancer [48]. Currently, further research is needed to explore the distinct characteristics of hTERT DNA methylation in different subtypes and stages of breast cancer.

#### hTERT Histone mark modifications

Histone mark modification is a significant epigenetic regulatory mechanism. In human chromatin, a segment of approximately 145 to 147 bp of DNA wraps around a histone octamer composed of two copies each of H2A, H2B, H3, and H4. This octamer, along with histone H1 located outside, forms a nucleosome, which repeats to create chromatin, resulting in highly compacted DNA within the cell [49]. A large number of epigenetic marks, including methylation, acetylation, and so on, can be found in the tails of histone proteins [50]. These marks play a role in influencing chromatin accessibility and subsequently impact DNA expression. Active histone marks commonly observed in cancer cells include dimethyl-H3K4, trimethyl-H3K4, acetyl-H3, acetyl-H4, and acetyl-H3K9, among others. Conversely, inactive histone marks include trimethyl-H3K9, trimethyl-H3K27, and others [51, 52] (Fig. 1C).

Recent studies have highlighted the significance of histone mark modifications, particularly methylation, and acetylation, in regulating hTERT expression in breast cancer cells. For instance, the compound genistein has been shown to increase the trimethyl-H3K9 mark associated with inactive histones, while decreasing the dimethyl-H3K4 mark associated with active histones within the hTERT promoter. These findings suggest that the inhibitory effect of genistein on hTERT expression in breast cancer cells is likely attributed, at least in part, to its influence on histone methylation patterns [42]. Sulforaphane can also change the methylation and acetylation patterns of chromatin histone in breast cancer cells. The change of histone modification promotes the binding of transcription repressors-CTCF and MAD1 to the regulatory region of hTERT and inhibits the binding of transcription activator c-MYC. As a result, it inhibits the expression of hTERT [44]. In another study, EGCG can alter histone methylation and acetylation patterns in breast cancer cells and regulate the binding of transcription factors through a mechanism similar to that of sulforaphane. Thereby, it affects the binding of MAD1 and c-MYC to the hTERT promoter and inhibits the transcription of hTERT [46]. Besides, centchroman treatment down-regulated the expression level of histone deacetylase (HDAC). In the meantime, the active markers acetyl-H3, acetyl-H4, and acetyl-H3K9 at the hTERT promoter were upregulated, while the inactive marker [53] trimethyl-H3K27 was downregulated [54]. This suggests that downregulation of HDAC is likely to result in decreased histone deacetylation of hTERT promoter, reduced marks of active acetylation, and thus decreased hTERT expression. The above studies have observed simultaneous regulation of hTERT DNA methylation and histone modification, indicating a potential interplay between these two epigenetic mechanisms in breast cancer cells. The interplay between histone mark modifications and DNA methylation has been extensively studied [55]. However, there are very few studies on the interaction between methylation and histone modification at the hTERT promoter. Delphine Garsuault et al. proposed an interplay between hTERT promoter methylation, chromatin accessibility, and histone modifications in acute promyelocytic leukemia cells, shedding light on the complex relationship between these epigenetic mechanisms [56]. There is also the interplay between hTERT promoter mutations and epigenetic modifications in hTERT expression, as shown by Catarina Salgado et al. [57]. Considering these findings, it is highly likely that similar interplay effects are present in breast cancer cells, highlighting the necessity for further investigation in this context.

#### micro-RNA

**Fig. 2:**
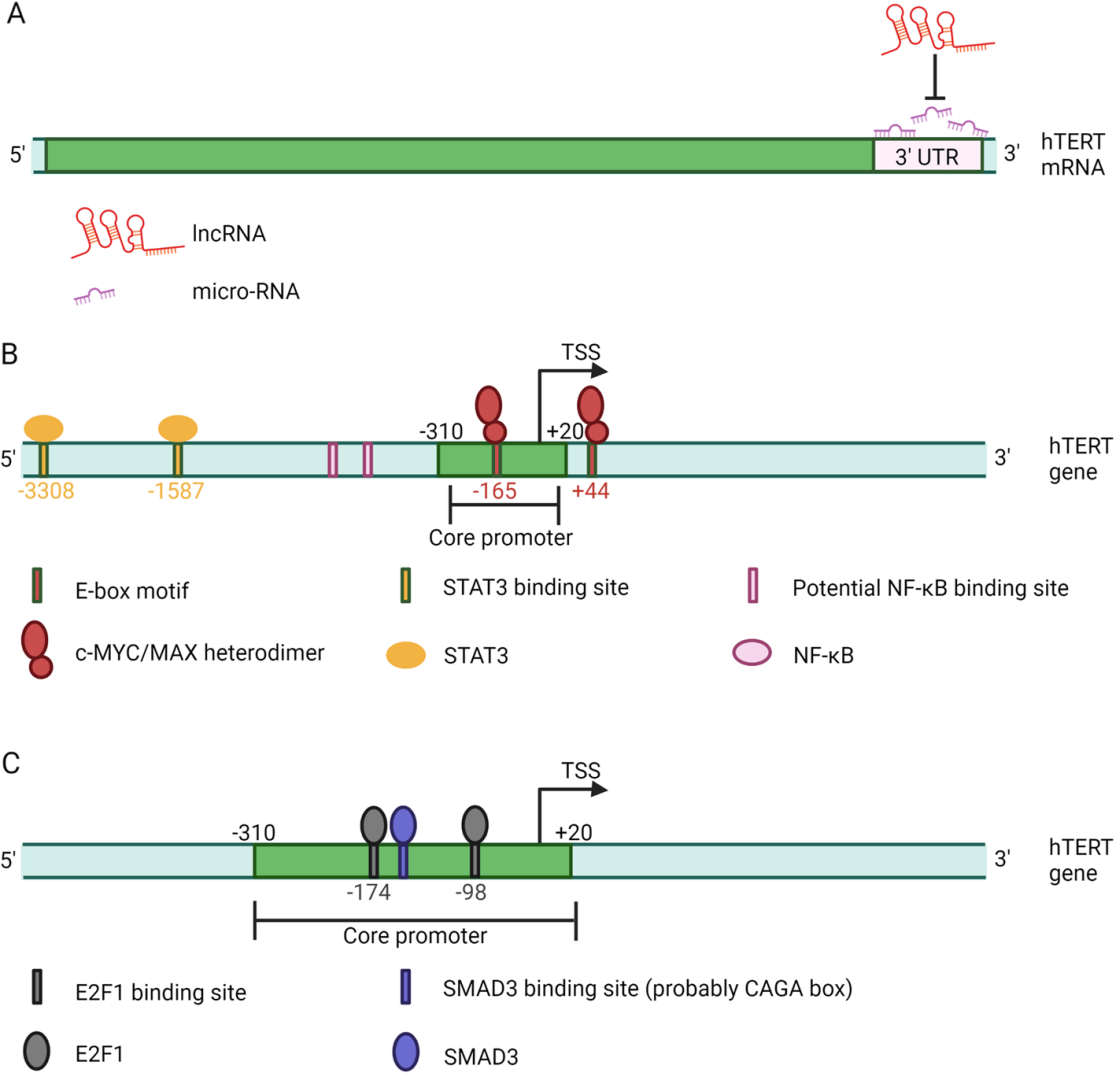
**A** Regulation of hTERT by micro-RNA, lncRNA and ceRNA. Micro-RNAs bind to the 3' untranslated region (UTR) of hTERT mRNA and silence the transcript. lncRNAs bind to micro-RNAs and prevent micro-RNAs from binding to hTERT mRNA. **B** Regulation of hTERT by transcriptional activators. c-MYC/MAX heterodimers bind to two E-box sites at -165 and + 44 nt of TSS. STAT3 binds to two STAT3 binding sites at -3308 and -1587 bp of TSS of hTERT promoter. NF-kB binds to two potential NF-kB binding sites at -664 to -654 and -758 to -749 bp of TSS. These transcriptional activators activate the hTERT promoter. **C** Regulation of hTERT by transcriptional inhibitors. E2F1 binds to two E2F1 binding sites at -174 and -98 bp of TSS. SMAD3 interacts with potential SMAD3 binding sites (probably a CAGA box which is adjacent to a MYC binding E-box). These transcriptional inhibitors inhibit the hTERT promoter. Created with BioRender.com

Non-coding RNA, including micro-RNA and lncRNA, plays a crucial role in the post-transcriptional regulation of hTERT expression and represents an important aspect of epigenetic regulation. Although less explored, it holds significant relevance. Micro-RNA is a small non-coding RNA molecule of 19–24 nucleotides in length [58]. Typically, it binds to the 3' untranslated region (UTR) of mRNA. This binding event leads to mRNA silencing and subsequently reduces gene expression (Fig. 2A). Micro-RNAs directly targeting hTERT mRNA have been shown to cause decreased hTERT mRNA expression, while micro-RNAs targeting other mRNAs may indirectly regulate hTERT expression. Consequently, micro-RNAs can function as either tumor suppressors or drivers, exerting a notable influence on hTERT expression and overall cellular processes.

Several micro-RNAs have been identified as regulators of hTERT expression in breast cancer. miR-296-5p and miR-512-5p expression are reduced in human breast cancer. What is more, the epigenetic silencing of miR-296-5p and miR-512-5p promotes hTERT expression in basal-type breast cancer cells, which is associated with a poorer prognosis [59]. Furthermore, miR-4458 directly targets the 3'-UTR of CPSF4. In breast cancer tissues and cell lines, the low expression of miR-4458 leads to increased CPSF4 expression, consequently resulting in upregulated hTERT expression [60]. Additionally, in estrogen-sensitive ovarian, endometrial, and breast cancer cells, the treatment with 1,25-(OH)2-D3 induces the upregulation of miR-498 and the downregulation of hTERT. This process antagonizes the stimulatory effect of leptin on hTERT expression and cell proliferation, revealing a regulatory role of miR-498 in hTERT-related pathways [61].

#### lncRNA and ceRNA

Apart from their interactions with mRNA, lncRNAs can also bind to micro-RNAs, preventing micro-RNAs from binding to their intended target mRNAs. This phenomenon is known as competing endogenous RNAs (ceRNA) [62]. When a lncRNA molecule competes with hTERT mRNA for binding to the same micro-RNA, it counteracts the inhibitory effect of the micro-RNA on hTERT gene expression (Fig. 2A). Consequently, the expression of hTERT in cancer cells is promoted. It is noteworthy that, besides lncRNAs, other non-coding RNAs, pseudogenes, and even mRNAs can also regulate the expression of target genes by targeting their corresponding micro-RNAs. These interactions create a complex and intertwined regulatory network referred to as the ceRNA network (ceRNET) [63].

The ceRNA network (ceRNET) has emerged as a pivotal player in the occurrence and progression of breast cancer [63], but only two published studies have focused on its relevance to hTERT. Cheng Li et al. found that miR-125a-3p exerts direct targeting effects on hTERT in breast cancer cells, along with CYP4Z1 and the pseudogene CYP4Z2P [64, 65]. The authors further confirmed that the ceRNET formed between CYP4Z1 and the pseudogene CYP4Z2P serves as a sub-ceRNET specifically dedicated to hTERT, as it competitively binds to miR-125a-3p, consequently inhibiting apoptosis in breast cancer cells [65]. Yujuan Kang et al. found that lncRNA SNHG1 acts as a ceRNA to promote hTERT expression by binding to miR-18b-5p in breast cancer cells [66]. It is highly probable that additional ceRNAs play potential roles in the regulation of hTERT expression, which requires further investigation.

### Transcription factors

Transcription factors, which are protein molecules, exert their regulatory function by binding to specific regulatory sequences within DNA, thereby either activating or inhibiting transcription. The function of transcription factors involved in the regulation of hTERT is extensive, and their interactions with each other are complex and widespread. Moreover, many of these transcription factors serve as downstream effectors within various signaling pathways and play pivotal roles in signal transduction.

#### Transcriptional activators

Several transcriptional activators have been identified to regulate hTERT expression in breast cancer cells, including c-MYC, STAT3, NF-κB, ETS2, ERα, Sp1, KLF4, and ZEB1/YAP, among others. Among these factors, c-MYC has been extensively investigated and represents the most studied transcriptional activator associated with hTERT regulation in breast cancer.

The c-MYC gene, an essential proto-oncogene belonging to the MYC gene family [67], forms a heterodimer with the MAX protein upon binding. The N-terminal transactivation domain of c-MYC recognizes E-box motifs (5'-CACGTG-3' sequence) on DNA, enabling c-MYC/MAX heterodimers to bind to regulatory sequences like promoters and regulate target gene transcription [68, 69]. Within the hTERT promoter, there are two E-box sites located at -165 and + 44 nt of the transcription start site (TSS) [70]. The c-MYC/MAX dimer exhibits specific binding to these two E-box sites on the hTERT promoter with equal probability [71] (Fig. 2B). Therefore, it activates the hTERT promoter. Moreover, c-MYC/MAX also contributes to the maintenance of chromatin-mediated hTERT gene repression [70]. Vassilis Papanikolaou et al. found a correlation between upregulated hTERT expression in HER-2 positive cells and the preferential binding of c-MYC/MAX complexes to the proximal E-box within the hTERT gene promoter, potentially mediating radiation resistance in HER-2 positive cells [72]. Treatment with pterostilbene simultaneously reduced the expression levels of c-MYC and hTERT in breast cancer cells, indicating that pterostilbene may downregulate hTERT expression in these cells by inhibiting c-MYC. Consequently, this leads to cell cycle arrest, apoptosis, and inhibition of cell proliferation [73]. In the context of previous studies, gefitinib, an inhibitor of epidermal growth factor receptor (EGFR) tyrosine kinase activity, was found to induce cell apoptosis. In MDA-MB-231 cells expressing high levels of EGFR, gefitinib treatment resulted in decreased expression of c-MYC, reduced DNA binding activity, and telomerase activity. Furthermore, gefitinib regulated the expression of various genes associated with the mTOR pathway [74], likely attributed to the interaction between the PI3K/Akt/mTOR pathway and c-MYC [75, 76]. Another study demonstrated that harmine treatment led to significant downregulation of both hTERT and c-MYC [77]. These findings suggest that the mechanism underlying the prevention of MCF-7 cell growth and induction of cell senescence by harmine is most likely associated with the inhibition of c-MYC expression, consequently suppressing telomerase activity.

STAT3, a member of the signal transducer and activator of the transcription (STAT) family, is an oncogene that plays a crucial role in cancer initiation and progression [78]. STAT3 promotes the expression of hTERT in human cancer and primary cells [79]. Within the hTERT promoter, there exist two binding sites for STAT3 located at -3308 and -1587 base pairs from the transcription start site (TSS). These binding sites enable direct interaction between STAT3 and the hTERT promoter, leading to the transcriptional activation of hTERT [80] (Fig. 2A). In addition, c-MYC is an important target for STAT3 to exert oncogenic effects [81]. In MDA-MB-231 cells, RIN1 has been shown to inhibit hTERT expression, potentially attributable to the downregulation of c-MYC, Ets2, and STAT3 activities [82]. In MCF-7 cells, leptin enhances the interaction between STAT3 and the hTERT promoter, thereby resulting in the upregulation of hTERT transcription [83].

Upon activation, NF-κB translocates into the nucleus and binds to specific binding sites on the target gene's promoter or enhancer, thereby initiating gene transcription [84]. NF-κB family members are highly expressed in various tumor types, including breast cancer, and exert regulatory control over cell cycle progression and apoptosis [85]. Within the hTERT promoter, two putative NF-κB binding sites have been identified (-664 to -654 and -758 to -749) (Fig. 2A), and their nucleotide sequences are very close to the consensus NF-kB binding site [86]. After irradiation, NF-kB is upregulated in HER2-positive breast cancer cells. Consequently, NF-κB binds to the promoter of c-MYC, inducing its expression, which subsequently leads to preferential binding to the hTERT promoter and increased hTERT expression [87]. Treatment of breast cancer cells with luteolin inhibited the NF-κB pathway and subsequent c-MYC expression, which significantly reduced hTERT expression levels [88]. This serves as an additional illustration of NF-κB's influence on c-MYC, indirectly modulating hTERT expression. Furthermore, an interplay between the NF-κB pathway and STAT3 has been observed, as they collaboratively participate in the regulation of hTERT expression in breast cancer cells [80].

In addition, ETS2 [89]、ERαand Sp1 [90], KLF4 [91], and ZEB1/YAP [92] have also been identified as regulators of hTERT transcription in breast cancer cells.

#### Transcriptional Repressors

Alongside the activators, several transcriptional repressors have been implicated in the regulation of hTERT expression in breast cancer cells. These include E2F1, Smad3, CTCF, YY1, and others.

E2F1, a member of the E2F family, plays a dual role in cellular processes. It acts as a promoter of cell proliferation and is closely associated with the transition from G1 to S phase of the cell cycle [93]. Additionally, E2F1 can also induce cell apoptosis and inhibit tumorigenesis [94]. Within the hTERT promoter, two E2F1 binding sites have been identified at -174 and -98 base pairs from the transcription start site (TSS). The binding of E2F1 to these sites leads to the inhibition of hTERT transcription [43] (Fig. 2C). Notably, E2F1 exhibits extensive interplay with c-MYC, and it can negatively modulate the impact of c-MYC on hTERT transcription [95]. In the context of genistein treatment, the downregulation of telomerase activity is accompanied by increased binding of E2F1 to the hTERT promoter [42]. Similarly, upregulation of E2F1 expression in 4T1 breast cancer cells following static magnetic field treatment has been observed to downregulate hTERT expression and reduce telomerase activity [96]. Moreover, E2F1 can indirectly regulate hTERT expression by binding to the promoters of other genes. For instance, in breast cancer, E2F1 binds to the SNHG1 promoter and enhances SNHG1 transcription, which in turn promotes hTERT expression by sponging miR-18b-5p [66]. The expression levels of E2F1 and c-MYC are often co-regulated. For example, aloe emodin downregulates hTERT transcription in three breast cancer cell lines by upregulating E2F1 expression and downregulating c-MYC expression [47].

SMAD3, a member of the SMAD family, functions as a downstream molecule in the TGF-β superfamily signaling pathway [97]. SMAD proteins regulate gene transcription by recognizing SMAD-binding elements and binding to specific DNA sequences [98]. Upon activation by TGF-β and recruitment by c-MYC, SMAD3 interacts with specific sites, potentially a CAGA box adjacent to an MYC binding E-box, within the hTERT promoter, leading to significant repression of hTERT gene transcription [97] (Fig. 2C). Furthermore, SMAD3 can antagonize the effect of c-MYC [97, 99]. In a study, bone morphogenetic protein-7 induces SMAD3 activation, facilitating its nuclear translocation and gene transcriptional activity. Consequently, hTERT activity is downregulated and telomeres are shortened in breast cancer cells [100].

In addition to E2F1 and SMAD3, several other transcription factors have been identified to down-regulate hTERT transcription in breast cancer. For instance, CTCF binds to exon 1 of the hTERT gene and inhibits its expression in breast cancer cells [44, 45]. Another example is YYI, which binds to the hTERT promoter in breast cancer stem cells upon NMI stimulation, leading to the down-regulation of hTERT expression [101].

### Single nucleotide polymorphism

**Fig. 3:**
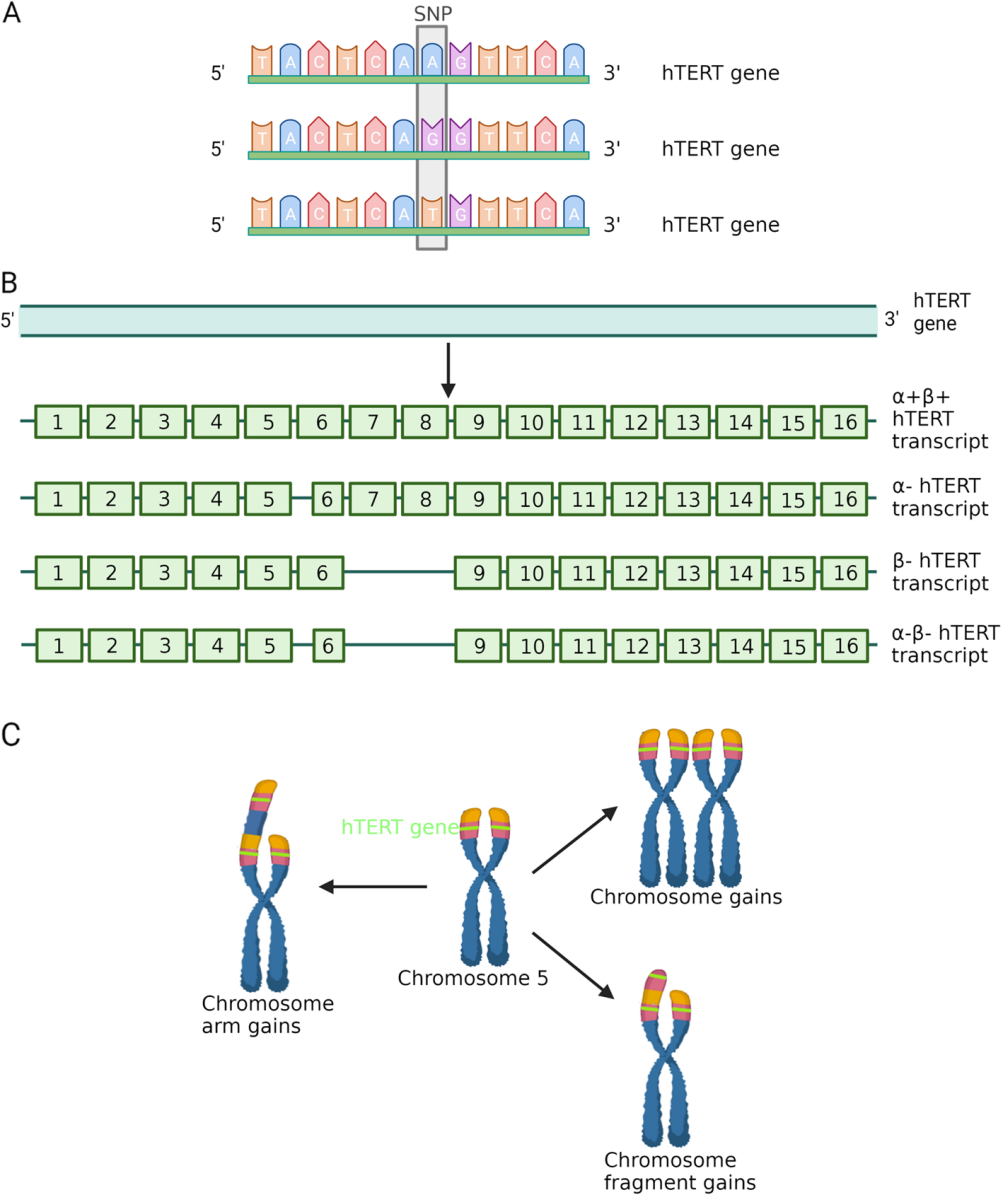
**A** Single nucleotide polymorphism of hTERT gene. Single nucleotide variation causes DNA sequence diversity of hTERT and affects its activity. **B** Alternative splicing of hTERT. The hTERT gene contains 16 exons and 15 introns. Alternative splicing of hTERT mainly produces three kinds of transcripts, namely α- (partial deletion of exon 6), β- (deletion of exons 7 and 8), and α-β-(deletion of exon 7 and 8, and partial deletion of exon 6). Only α + β + full length hTERT transcripts can lead to telomerase activity. **C** hTERT Copy Number Amplifications. hTERT is located on the short arm of chromosome 5 (5p). Chromosome fragment, chromosome arm or chromosome gains can lead to amplifications of hTERT gene. Created with BioRender.com

Single nucleotide polymorphisms (SNPs) are genetic variations caused by a single nucleotide change in the DNA sequence at the genome level (Fig. 3A). SNPs can occur in both coding and non-coding regions [102]. Numerous studies have indicated that SNPs are associated with the susceptibility and recurrence of cancer [103–105].

A meta-analysis revealed that certain sites within the hTERT gene and the adjacent CLPTM1L region were linked to the risk of multiple cancers. Specifically, the rs75316749 variant in the TERC region was positively associated with colorectal, breast, ovarian, and lung cancers. In the DCLRE1B region, the rs974404 and rs12144215 variants were inversely associated with prostate, lung, colorectal, breast, and ovarian cancers, respectively [106]. Another meta-analysis conducted by Zhang et al. identified 15 common genetic variants significantly associated with breast cancer risk, with the most significant association observed for a non-coding variant (hTERT rs2853669) [107]. The minor allele of rs2736109 and another hTERT promoter SNP, rs2736108, were associated with reduced breast cancer risk, and the combination of these two SNPs led to a substantial reduction in hTERT promoter activity [108]. Haiman et al. found that the rs10069690 variant at the hTERT-CLPTM1L site on chromosome 5p15 was associated with the risk of ER-negative breast cancer, particularly in younger women (< 50 years old), and also showed a significant association with triple-negative breast cancer [109].

In terms of monoallelic risk analysis, a significant association was observed between the hTERT rs2853676 allele A and cancer susceptibility. However, stratified analyses revealed increased cancer risk in the glioma, lung, and ovarian cancer subgroups, but not in the breast cancer subgroup [110]. Additionally, Liu et al. found no association between the hTERT rs2853669 polymorphism and breast cancer risk [111]. Further studies are needed to gain a better understanding of the role of SNPs in breast cancer.

### Alternative splicing

Alternative splicing is a process that generates different mRNA transcripts from the same gene, leading to the production of diverse protein isoforms [112]. The hTERT gene, consisting of 16 exons and 15 introns, undergoes alternative splicing by including or excluding specific exons during mRNA processing [113]. Notably, three sites, referred to as α (exon 6), β (exons 7 and 8), and γ (exon 11), are commonly subject to splicing events in hTERT [114, 115]. Splice variants lacking specific exons, such as α and β, result in the loss of telomerase activity [115]. Among these variants, α, and β are the most extensively studied [116] (Fig. 3A). For instance, the deletion of the α site (hTERT α-) has been shown to lead to shortened telomeres by reducing telomerase activity, thereby limiting cell growth potential [117].

In breast cancer, hTERT is highly expressed in most cases [118]. The β-deletion splice variant accounts for the highest proportion of hTERT transcripts in a panel of breast cancer cells [115, 119]. The splicing regulators SRSF11, HNRNPH2, and HNRNPL control the splicing of this transcript, and its presence is associated with polysomes in cells. Higher telomerase activity is observed in basal subtype breast cancer cells, which exhibit a higher expression ratio of full-length α + β + transcripts. In contrast, the luminal subtype shows lower telomerase activity, accompanied by higher levels of β-deletion variants. Therefore, the isoform-specific splicing of hTERT is linked to variations in telomerase activity among different breast cancer subtypes [115].

The alternative splicing of hTERT, including full-length and β variants, has an impact on telomerase activity in breast cancer. While the presence of full-length variants is important, it is not sufficient to promote telomerase activity when other splicing variants are abundant simultaneously [120]. Forced overexpression of β-deleted protein inhibits endogenous telomerase activity [119]. Moreover, the β-deletion variant has been detected in both the nucleus and mitochondria, offering protection against cisplatin-induced apoptosis in breast cancer cells [115].

It has been found that celthrin, when present at a high concentration, can alter the splicing pattern of hTERT in MCF7 breast cancer cells. This leads to the production of a non-enzymatic coding isoform of the hTERT transcript, consequently inhibiting the total transcription and activity of the full-length variant of hTERT. This mechanism potentially contributes to the telomerase activity inhibition observed in MCF7 cells upon celthrin treatment [121].

### hTERT copy number amplifications

Chromosome gains and losses, including those involving the hTERT gene located on chromosome 5, are frequently observed in solid tumors [122] (Fig. 3C). A 5p gain is detected in approximately 13.2% of solid tumors, indicating a common occurrence [122]. Notably, additional amplification of hTERT is often associated with highly malignant tumors and a high risk of recurrence [123]. Studies have shown a positive correlation between increased copy numbers of hTERT, hTERT expression, and telomerase activity in tumor cell lines. This relationship has also been established in breast and lung cancer tumor samples [124].

In metastases from patients with triple-negative breast cancer, hTERT amplification has been detected, along with amplifications of AKT2 and CCNE1 [125]. Moreover, hTERT promoter hotspot mutations and gene amplification have been identified in malignant breast cancers with predominantly non-chondroid components, accounting for 17% of cases [18]. Considering the presence of these alterations in both breast cancer and breast malignant phyllodes tumors, we should be more cautious when using hTERT promoter hotspot mutations and gene amplification for differential diagnosis between these two diseases [18].

### Posttranslational Modification of hTERT

**Fig. 4:**
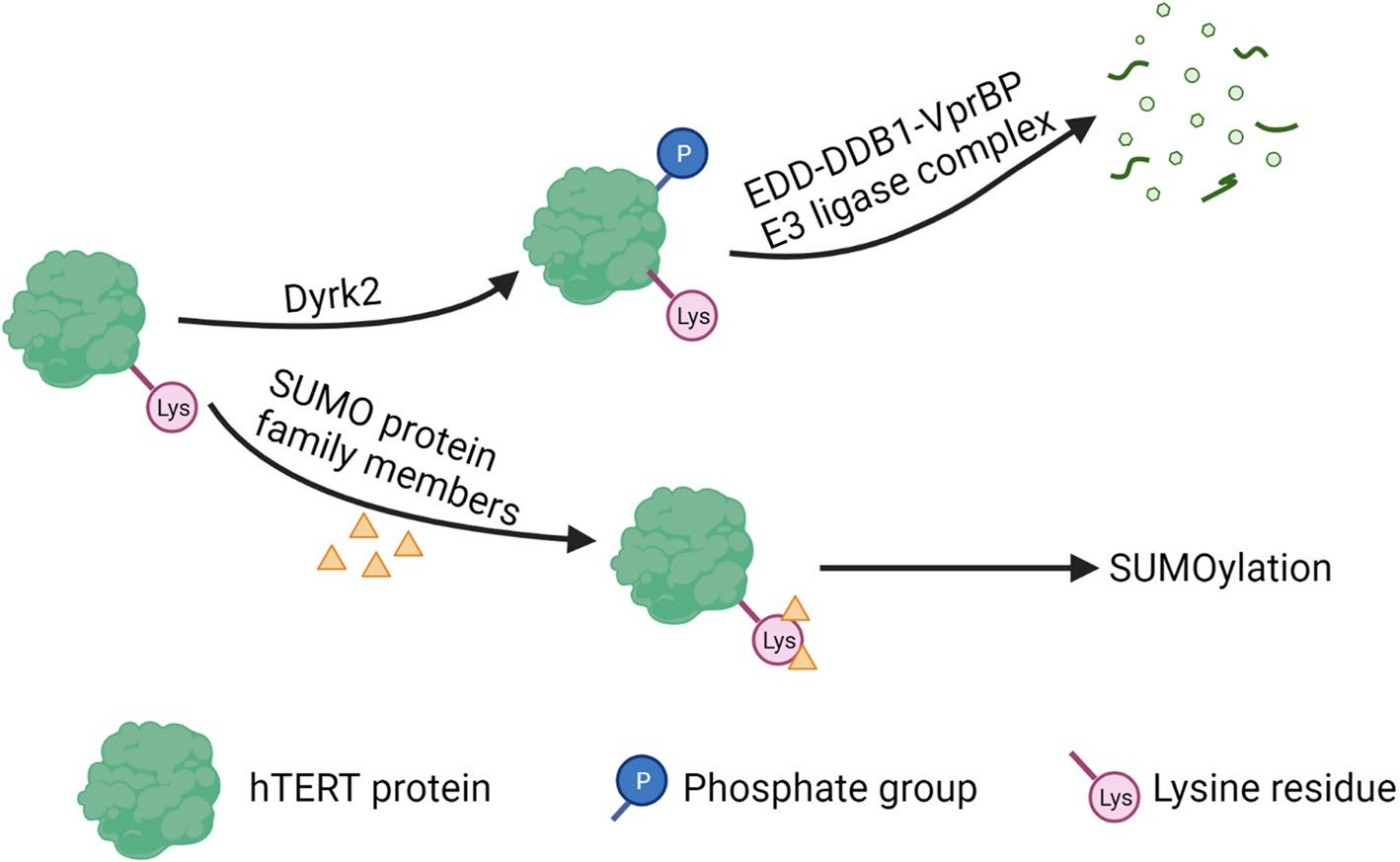
Posttranslational modification of hTERT. In the ubiquitin-mediated degradation process of hTERT, Dyrk2 phosphorylates hTERT protein. Then, phosphorylated hTERT binds to the EDD-DDB1-VprBP E3 ligase complex and the protein is degraded. In the SUMOylation process, members of the small ubiquitin-like modifier (SUMO) protein family bind to lysine residues in hTERT protein and upregulates hTERT activity. This process is also catalyzed by enzymes. Created with BioRender.com

Posttranslational modification plays a crucial role in regulating the abundance and function of the hTERT protein. One important form of modification is ubiquitination, which involves the attachment of ubiquitin molecules to substrate proteins through a cascade of enzymes including E1s, E2s, and E3s [126]. Ubiquitination has a variety of functions. The most important one is to lead to the degradation of substrate proteins. Dyrk2 phosphorylates hTERT, enabling its binding to the EDD-DDB1-VprBP E3 ligase complex and subsequent ubiquitin-mediated degradation of the TERT protein (Fig. 4). Dyrk2 S471X nonsense mutation identified in breast cancer leads to elevated hTERT expression in HeLa cells [127]. This suggests that post-translational phosphorylation and ubiquitination of hTERT may also regulate its protein levels in breast cancer cells. Another example of post-translational modification is the interaction between hTERT and the ubiquitin-conjugating enzyme E2D3 (UBE2D3). Inhibition of UBE2D3 expression results in hTERT accumulation in MCF-7 cells, thereby reducing radiosensitivity and promoting cell proliferation [128]. This highlights the significance of ubiquitination as a post-translational modification of hTERT.

Additionally, members of the small ubiquitin-like modifier (SUMO) protein family can bind to lysine residues in target proteins, a process known as SUMOylation [129] (Fig. 4). SUMOylation represents another form of post-translational modification of hTERT. CBX4 acts as a SUMO E3 ligase, leading to SUMOylation of hTERT and upregulation of its activity, consequently promoting migration and invasion of breast cancer cells [130].

Furthermore, Akt activation can increase telomerase activity by phosphorylating the hTERT protein [56]. In a study, gefitinib treatment downregulated phosphorylated Akt, hTERT phosphorylation, and nuclear translocation of hTERT in MDA-MB-231 cells [42]. Additionally, phosphorylation of hTERT at threonine 249 has been associated with aggressive features in various cancers, including triple-negative breast cancer [131].

## The function of TERT in breast cancer

### Canonical function

Tumor cells achieve immortality through two mechanisms: the telomere-maintenance mechanism (TMM) and the alternative lengthening of telomeres (ALT). TMM, which relies on telomerase, is present in 80–90% of tumor cells [132]. ALT, on the other hand, occurs in 10–15% of tumors, particularly those of neuroepithelial or mesenchymal origin [133–137]. ALT is associated with a higher degree of malignancy compared to TMM. Since TMM is the main regulatory strategy in breast cancer, ALT will not be described in detail in this review.

Telomerase, a ribonucleoprotein enzyme composed of TERC and hTERT subunits, plays a crucial role in maintaining telomere length. It utilizes reverse transcriptase activity to synthesize new telomere DNA, allowing tumor cells to maintain telomere activity even when they become critically short [138]. In the study by Syed M Meeran et al., the mechanism of sulforaphane (SFN) in inhibiting the viability and proliferation of breast cancer cells was investigated. The findings revealed that SFN hindered hTERT expression through epigenetic pathways, leading to apoptosis of breast cancer cells [44]. Another study conducted by Karuvaje Thriveni et al. explored the relationship between hTERT gene expression and telomere length in breast cancer patient tissues. Their results indicated that 39% of patients with high hTERT expression exhibited telomere elongation, while 23% of patients with low hTERT expression displayed shorter telomeres. This study confirmed that hTERT can compensate for telomere attrition during tumor replication [139].

### Non-canonical Functions

Non-canonical functions of telomerase are those unrelated to telomere maintenance, often referred to as telomere-independent functions. It may involve the up-regulation of telomerase activity but does not lead to telomere elongation [140]. Besides its role in telomere maintenance, hTERT expression is also associated with these non-canonical functions [140]. In breast cancer, hTERT's non-canonical functions are primarily involved in regulating cell cycle progression, autophagy, senescence, apoptosis, signal transduction, transcription factors, epithelial-mesenchymal transition (EMT), cell adhesion, and migration.

In a study using MCF-7 cells, indole-3-carbinol (I3C) was found to significantly downregulate hTERT gene transcription and induce G1 phase cell cycle arrest. Additionally, I3C disrupted the interaction between estrogen receptor α (ERα), transcription factor Sp1, and the hTERT promoter, illustrating one mechanism by which hTERT expression can impact cell cycle progression [90].

hTERT also has inhibitory effects on cancer cell senescence, apoptosis, and autophagy. For instance, the cytokine bone morphogenetic protein-7 (BMP7) induces hTERT inhibition, leading to senescence and apoptosis in MCF-7 cells [100]. Breast cancer cells express not only intact α + β + hTERT transcripts but also highly express α + β- hTERT transcripts. These α + β- hTERT proteins competitively bind to TERC, inhibiting telomerase activity and protecting breast cancer cells from cisplatin-induced apoptosis [115], highlighting the importance of considering hTERT splice variants. A study by Aleksandra Romaniuk-Drapła et al. observed activation of autophagy in MDA-MB-231 cells following lentivirus-mediated downregulation of hTERT [141], providing supporting evidence for the inhibitory role of hTERT in autophagy.

hTERT exerts its effects on signaling pathways and transcription factors primarily through NF-κB and Wnt/β-catenin pathways, as well as transcription factors like c-MYC. Telomerase directly regulates NF-κB-dependent gene expression by binding to the p65 subunit of NF-κB [142], which in turn activates hTERT transcription. While there are numerous studies investigating the regulation of hTERT expression by NF-κB in breast cancer, the role of telomerase in modulating the NF-κB signaling pathway in breast cancer cells remains understudied. Nevertheless, it is likely that such a mechanism exists. Additionally, hTERT can influence Wnt/β-catenin signaling through its interaction with BRG1 [143], although research focusing on this aspect in breast cancer cells is limited. The interaction between c-MYC and hTERT is also noteworthy. Ectopic expression of pescadillo (PES1) enhances c-MYC expression in MCF-7 cells, with this effect being significantly reduced in TERT knockdown cells, suggesting that PES1 primarily promotes c-MYC expression through hTERT [144].

In addition to its involvement in telomere elongation, hTERT plays crucial roles in EMT, cell adhesion, and migration. A study has demonstrated that ectopic expression of hTERT up-regulates TSPAN13, which activates EMT and inhibits cancer cell apoptosis, thereby promoting proliferation and migration [145]. Moreover, hTERT promotes EMT by upregulating the urokinase plasminogen activator (uPA) through TGF-β signaling [146]. Additionally, hTERT enhances migration and invasion of breast cancer cells in a SUMOylation-dependent manner [130]. The down-regulation of hTERT by N-myc and STAT interactor (NMI) inhibits EMT and cell invasion in breast cancer cell lines [101]. Notably, hTERT plays a complex role in regulating EMT in breast cancer stem cells, suggesting the existence of a bidirectional feedback loop [147].

## The potential of hTERT as a diagnostic and prognostic marker for breast cancer

The high expression of hTERT in cancer cells and its involvement in telomere maintenance make it a potential diagnostic and prognostic marker for breast cancer. Detection of hTERT expression can aid in early screening, classification, prognosis analysis, and treatment selection.

Zhe-Yu H et al. observed an increased frequency of TERT mutations in circulating tumor DNA (ctDNA) from patients with HR + metastatic breast cancer who experienced disease progression within 3 months after multi-line therapy [148]. This suggests a potential association between hTERT and disease progression in HR + multi-line resistant metastatic breast cancer. Aleksandra Romaniuk-Drapała et al. demonstrated that the downregulation of hTERT increased the sensitivity of breast cancer cells to doxorubicin [141]. Studies on residual breast tumors following neoadjuvant chemotherapy indicated that shorter telomeres and higher hTERT levels were independently associated with worse survival [149]. Furthermore, a separate study reported high tissue hTERT expression in 63% of invasive breast cancer patients, with an odds ratio of 2.77 [139]. Mahendar Porika et al. found that serum hTERT levels were significantly higher in breast cancer patients compared to healthy individuals, with a sensitivity and specificity for breast cancer diagnosis of 68.9% and 83.3%, respectively. Moreover, the level of serum hTERT prior to treatment showed a significant correlation with the clinical stage [150]. Importantly, the cellular localization of hTERT may also influence drug resistance and disease progression in breast cancer. For instance, hTERT can dynamically translocate to the cytoplasm to confer resistance to HER2 subtype after primary systemic therapy [151].

Transcription factors and DNA methylation associated with hTERT offer valuable insights for breast cancer diagnosis and prognosis. The BRCT-repeat inhibitor of hTERT expression (BRIT1) has been found to inhibit hTERT transcription [152]. In familial breast cancer patients, high cytoplasmic and low nuclear expression of BRIT1 correlated with high histological grade. In sporadic breast cancer patients, cytoplasmic BRIT1 positivity was significantly associated with progesterone receptor positivity [153]. The NMI-YY1-hTERT pathway revealed a negative correlation between NMI expression and hTERT expression. Low NMI/high hTERT expression was linked to poor TNM stage [101]. Moreover, THOR demonstrated significant hypermethylation in malignant breast tissues compared to benign tissues, offering the potential for distinguishing malignant tumors from normal tissues in early-stage disease [48]. These findings provide promising avenues for future research.

Furthermore, single nucleotide polymorphisms (SNPs) in the hTERT gene have been found to impact the risk of breast cancer. For instance, rs10069690 was significantly associated with an increased risk of ER-negative and triple-negative breast cancer, particularly in younger women (< 50 years old). This may contribute to the higher incidence of ER-negative and triple-negative tumors in women of African descent compared to women of European descent [109]. Similarly, rs2853669 and rs2736109 were also associated with breast cancer risk [154, 155]. However, some studies have reported no association between certain SNPs and breast cancer risk [110, 111], emphasizing the need for further investigation in this area.

In recent years, several studies have employed RT-qPCR [156] to detect circulating tumor cells (CTCs) expressing TERT and other molecules in peripheral blood [157–160]. This approach aids in predicting prognosis and identifying patients at risk of metastasis at an early stage. These findings highlight the diverse potential applications of hTERT as a diagnostic and prognostic marker that warrant further research.

## Therapeutic potential of hTERT as a target for breast cancer

The exploration of hTERT's regulatory mechanism has opened up promising avenues for the treatment of breast cancer. Recent studies have focused on targeting hTERT and its expression and regulation processes through various approaches. These include the use of quadruplex-binding small-molecule ligands and other substances that can modulate hTERT expression through epigenetic mechanisms, alternative splicing, signaling pathways, and transcription factors. Additionally, potential therapeutic strategies such as small interfering RNA (siRNA) and hTERT vaccines are being investigated.

G-quadruplex (G4) is a secondary DNA helix structure formed by guanine-rich nucleic acids, which can be found in the promoters of multiple genes. By stabilizing the G4 structure, gene transcription can be inhibited [161]. Small-molecule ligands that bind to quadruplexes can stabilize the G-quadruplex structure at telomere ends, preventing telomerase access and impeding telomere elongation mediated by hTERT upregulation [162, 163]. Several quadruplex-binding small-molecule ligands have shown potential for breast cancer treatment, including BRACO19 [164], RHPS4 [165], TMPyP4 [163], BIBR1532 [166], etc. Among these, BIBR1532 has received significant attention in recent years. Studies have demonstrated its synergistic effects in combination with NK cell therapy [167], paclitaxel [168], and arsenic trioxide [169].

Therapeutic strategies aimed at epigenetic regulation have been shown to impact hTERT expression. Drugs targeting histone modifications and DNA methylation, such as centchroman [54], genistein [42], sulforaphane [44], and EGCG [46], have demonstrated their potential in modulating hTERT expression.

In the context of alternative splicing, chelidonine has been found to influence the splicing pattern of hTERT, favoring the production of non-enzyme coding isoform transcripts [121]. Additionally, ellagic acid has been shown to significantly reduce the expression of hTERTα + β + mRNA induced by 17β-estradiol in MCF-7 cells, potentially contributing to its chemopreventive effect on breast cancer [170].

Targeting transcription factors and signaling pathways that regulate hTERT represents another viable therapeutic approach. Notable examples include c-MYC [73, 171], PI3K/Akt, and TGF-β pathways [172], etc.

siRNA can effectively silence the hTERT gene post-transcriptionally [173]. Both low-dose adriamycin and hTERT siRNA demonstrate the ability to inhibit hTERT expression in breast cancer cells, exhibiting a synergistic effect [174]. Furthermore, the targeted co-delivery of doxorubicin and hTERT siRNA can effectively suppress hTERT expression [175].

Given the prevalent high expression of hTERT in cancer cells, therapeutic hTERT vaccines hold promise in eliminating cancer cells by enhancing the activity of telomerase-specific CD8 + T cells [176]. However, clinical trials involving breast cancer patients remain limited, with only a few studies conducted thus far [176, 177], and no exclusive trials focusing solely on breast cancer patients have been reported.

## Conclusions

The upregulation of hTERT in most cancer cells contributes to their telomere maintenance ability and high telomerase activity. In contrast, hTERT is not actively expressed in normal adult somatic cells. The regulatory mechanisms underlying hTERT overexpression in breast cancer are multifaceted, involving promoter mutations, epigenetic modifications, transcription factors, single nucleotide polymorphisms, alternative splicing, copy number amplification, and post-translational modifications. There is plenty of interplay between different mechanisms, and they could act simultaneously. Upregulated hTERT primarily promotes cancer cell survival and disease progression through telomere maintenance mechanisms. Moreover, in breast cancer, hTERT also modulates various cellular processes, including cell cycle regulation, apoptosis, autophagy, cell adhesion and migration, and cell signal transduction, independently of its role in telomere maintenance. Although the understanding of hTERT regulatory mechanisms has advanced, further research is needed to deepen our knowledge, particularly in certain regulatory mechanisms. Concerning the role of SNPs in particular, several studies have yielded different results. For example, the study by Zhengsheng Liu et al. concluded that rs2853669 was not associated with breast cancer risk [111], while Sonja Helbig et al. suggested that rs2853669 plays a role in TERT promoter regulation [178].

Given the high expression of hTERT in breast cancer, hTERT and its associated molecules hold promise as biomarkers for enhancing early screening and prognostic assessment of breast cancer. Detection methods typically involve examining hTERT and related molecules in tissues and serum. Additionally, exploring the cellular localization of hTERT and utilizing hTERT as a target for detecting circulating tumor cells in peripheral blood have shown advantages. Integration of hTERT into established panels may enhance sensitivity and specificity, which needs to be further studied.

In terms of treatment, various approaches are being explored, including drugs that stabilize the G-quadruplex structure, such as BIBR1532, as well as investigations into epigenetic regulation, alternative splicing, signal transduction, transcription factors, and siRNA-mediated hTERT gene silencing. Therapeutic hTERT vaccines also hold potential as a research direction. However, most of these studies remain at the laboratory stage, with only two clinical trials investigating therapeutic hTERT vaccines in breast cancer patients. Combining multiple treatments targeting hTERT and other mechanisms may yield synergistic effects. Further research in this area, particularly investigating combination therapies, is needed.

This review provides a comprehensive summary of recent studies on the role of hTERT in breast cancer, and its regulatory mechanisms, and explores the potential of hTERT as a biomarker and therapeutic target. A deeper understanding of these regulatory mechanisms and their interactions will contribute to the development of improved approaches for breast cancer treatment. Future advances in this field are eagerly anticipated. We look forward to seeing new advances in this field.

## Data Availability

Not applicable.

## Abbreviations

TERT/hTERT: Telomerase Reverse Transcriptase
TERC: Telomerase RNA
ALT: Alternative lengthening of telomeres
TERTp: HTERT promoter
MBCs: Metaplastic breast cancers
DNMT: DNA methyltransferase
5mC: 5-Methyl cytosine
TSS: Transcription start site
THOR: TERT hypermethylated oncological region
HDAC: Histone deacetylase
UTR: Untranslated region
ceRNA: Competing endogenous RNAs
ceRNET: CeRNA network
EGFR: Epidermal growth factor receptor
STAT: Signal transducer and activator of transcription
UBE2D3: Ubiquitin-conjugating enzyme E2D3
SUMO: Small ubiquitin-like modifier
TMM: Telomere-maintenance mechanism
sulforaphane: SFN
EMT: Epithelial-mesenchymal transition
I3C: Indole-3-carbinol
ERα: Estrogen receptor α
BMP7: Bone morphogenetic protein-7
PES1: Ectopic pescadillo
NMI: N-myc and STAT interactor
ctDNA: Circulating tumor DNA
BRIT1: BRCT-repeat inhibitor of hTERT expression
SNPs: Single nucleotide polymorphisms
CTCs: Circulating tumor cells
G4: G-quadruplex
siRNA: Small interfering RNA
